# The Hospital. Nursing Section

**Published:** 1907-02-23

**Authors:** 


### The Hospital.

**Nursing Section.**

Contributions for "THE HOSPITAL," should be addressed to the EDITOR, "THE HOSPITAL" NURSING SECTION, 28 & 29 Southampton Street, Strand, London, W.C.

No. 1,CG7.—VOL. XLI. SATURDAY, FEBRUARY 23, 1907.

### Notes on News from the Nursing World.

### NURSES ON BATTLESHIPS.

THE employment of female nurses on battleships is a very different thing from their employment on hospital ships accompanying the fleet in action. If, unhappily, this country were engaged in a naval war, we are sure that the services of female nurses would be indispensable on hospital ships. But their presence on battleships, in the first fighting line, which has been proposed, would be much more embarrassing than useful. It is incredible that such a proposal would receive the sanction of the authorities; more especially as naval orderlies—the Sick Berth Staff as they are called—are now receiving a good training in nursing. They are trained at the naval hospitals at Portsmouth and Plymouth, much of the instruction being given in the wards by the naval nursing sisters.

### NURSING AS A LIVELIHOOD.

THE matron of Charing Cross Hospital has been explaining to the representative of a lay paper the difference between the nurses of yesterday and to-day. Miss Heather-Bigg denies that when she became a nurse, twenty-two years ago, they were bad old times. She thinks that the nurses of those days were not more tired, in spite of longer hours and less recreation, than the nurses of to-day; and she believes that at that period women usually took up nursing because they felt that the life was a vocation. Now, she is of opinion that the motive in most instances is to earn a living, but we gather that she is indisposed to blame anyone for looking upon it in that light. With all our admiration for the still numerous women who regard nursing as a vocation, we are unable to see why it should not also be regarded as a mode of gaining a livelihood. In the ideal nurse the two motives are nicely balanced and thus zeal is tempered by discretion.

### AN APPEAL TO THE CENTRAL AUTHORITY.

THE Northallerton Guardians having refused to comply with the request of Dr. Hutchinson, their medical officer, to provide a night attendant for the workhouse, the latter, we think, took the right action in deputing the matter to the Local Government Board. Having made it clear to the Guardians that a night attendant is necessary for the cases which are at present in the infirmary, he was not only justified, he was bound in the interests of the sick poor, to appeal to the central authority. The Local Government Board have now asked the Guardians for an explanation, and they have referred the question to the Finance Committee to consider and report thereon. The worst of it is, that while the Guardians are shilly-shallying about, some of the patients in the infirmary, without a night attendant, may suffer from their inaction, even to the extent of losing their lives.

### AN OPENING FOR PRELIMINARY TRAINING.

IT will be observed, from the report of our Commissioner's interview with the matron of the Alexandra Hospital for Children with Hip Disease, that a new rule for the admission of probationers has just come into operation, and that the course of instruction, which was formerly for one year, now extends over two. The training is purely of a preliminary character, and is chiefly intended for young women who are not old enough to enter a general hospital but desire a useful occupation. The matron states that she does not think that the probationers leaving the institution experience difficulty in obtaining training elsewhere, and they should, at any rate, possess a good knowledge of the nursing of helpless children who need the maximum of care and attention.

### INCREASE OF STAFF AT BIRKENHEAD INFIRMARY.

QUICKLY following the appointment of the new assistant superintendent nurse at Birkenhead Workhouse Hospital, the Guardians have unanimously decided to engage three additional nurses for night duty. The need for the increase was clearly shown. For example, the guardian who moved the resolution in favour of the addition stated that on one occasion only a single probationer had been on night duty in charge of eleven wards. We are glad that there was no opposition to the motion, and that the Birkenhead Guardians are doing their best to level up the nursing at the Workhouse Hospital, instead of falling back upon the plea that other institutions are content to be behind the times.

### A STAFFORDSHIRE SCANDAL.

THE report in the lay Press that the Hanley, Stoke, Fenton, and Longton Joint Hospital Board have instructed their Clerk to prosecute a man, an ex-patient, who got over the wall of the Isolation Hospital two miles from Hanley, in order to see one of the nurses, is not correct. As a matter of fact, the Board are unable to prosecute because the patient who gave the information to the matron and charge nurse refuses to make a statement in court. Having obtained access to the building the man in question went in the night to the ward where this nurse was on duty, paying his visits between 12 and 2 A.M., and has, we understand, written a letter to the matron confessing his visits. The nurse to whom he refers denies having seen him, but we think, under the circumstances, the Board did well to approve the action of the medical officer in discharging her.

### GRATITUDE FOR SMALL MERCIES.

In the annual report of the Trained Nurses' Annuity Fund for Disabled Nurses, just issued, it is mentioned, with gratitude, that the general subscriptions last year show an increase, and that there are now fifteen annuitants. In other words, the fund, after being established for thirty-two years, is able to grant annuities to the amount of £255. Of course, £17 a year is very much better than nothing, but that there should be but fifteen worn-out trained nurses in receipt of this pittance is a discouraging outcome of the efforts made by the Council. The extracts from the letters received by the hon. secretary in 1906 are pitiful in the extreme; the more so from the fact that the Council could not vote more than a single additional annuity. We are glad to learn that Miss Sidney Browne has recently joined the Council and hope much from her well-known energy.

### TRAINING AT BELFAST INFIRMARY.

A proposal by the Belfast City Corporation that the Belfast Guardians should supply them with probationers for training in the Infectious Diseases Hospital at Purdysburn, has lately been agreed to. But a further suggestion of the Infirmary Committee, that two vacancies in the nursing staff of the Infirmary should be filled by temporary appointments, was set aside, and an amendment in favour of an inquiry into the nursing system was adopted instead. We think if, as one of the Guardians said, there are at present more than the number of nurses originally contemplated, this is a wise precaution. It is possible that in spite of this increase the number may be proved inadequate to meet the standard of a great Poor-law institution.

### DISTRICT NURSES AND BICYCLES.

The value of a bicycle to a district nurse has long been recognised. In fact it has become practically indispensable. It has also the great advantage of being very inexpensive. The Committee of the Leamington District Nursing Association, in appealing for funds to enable them to provide a new or second-hand bicycle for the use of their three nurses, mention that the one which was given them nine years ago is completely worn out. As a serviceable bicycle can be purchased for £10, this represents an expenditure of only a little more than £1 a year. But we think that the time has come when a bicycle should be recognised as part of the necessary equipment of a nurse engaged in district work, and paid for by the Association as a matter of course.

### A SHAMEFUL SLANDER.

At the Sussex Assizes on Saturday, an action for slander was tried, the plaintiff being Miss Woodward, a nurse. She had attended two patients at Brighton—Mr. and Mrs. Nash—and after the death of the latter she continued to nurse the former. The alleged slander was to the effect that the nurse had been guilty of immoral relations with her employer, that she had obtained possession of his wife's jewellery, and that she had been instrumental in causing the death of Mrs. Nash. These odious charges were all proved in court to be absolutely unfounded. Mr. Justice Bucknill having sharply interrogated Miss Nelson, the defendant, who was threatened with imprisonment for contempt of court before she would answer, she had to make the most serious admissions, and the jury then awarded the plaintiff £100 damages. We are glad that Miss Woodward had the courage to take the case into court, and congratulate her upon the vindication of her professional and personal character.

### GUARDIANS AND NURSING ASSOCIATIONS.

We record with satisfaction the fact that the Long Ashton Guardians have resolved, at the instance of Sir Henry Miles, to contribute five guineas a year to the support of the district nurse for the parish of Pill, and that the Bideford Guardians have decided upon increasing their yearly subscription to the Nursing Associations in their Union which extend their work to pauper patients. A most striking testimony of the soundness of this policy was given by one of the Bideford Guardians when the matter was discussed. He stated that, while the Board paid £23 last year for the nursing of the poor in Hartland, where the district nurse does not tend the sick in receipt of parochial relief, at Northam where such persons are included among the nurse's patients, the cost of nursing to the Board for the year was covered by the guinea subscribed to the Nursing Association. The Taunton Guardians, who have been asked to increase their subscription to the Kingston Association by a guinea, have postponed their decision, but in the light of the experience of the Bideford Board, we should think that the request will be acceded to. Meanwhile, the Chorley Guardians, unable to come to a definite conclusion, have resolved to make an annual grant of £15 to the District Nursing Association, subject to the approval of the Local Government Board.

### A GOOD EXAMPLE AT STAFFORD.

An excellent feature of the report of the Stafford District Nurses' Society is that the income is derived, not from large subscriptions from a few individuals, but from small sums from many. Last year the income was £254, as against an expenditure of £200, and although the balance in hand at the beginning of the twelve months was £48, the figures indicate that there has been a slight increase in contributions. The nurses attended more than 200 cases, and paid more than 5,000 visits. We congratulate the Committee upon the pains which are so successfully taken to obtain the regular support of the class which almost exclusively derives benefit from the work of the nurses.

### GUY'S HOSPITAL PAST AND PRESENT NURSES' LEAGUE.

A course of post-graduate lectures on the newest methods and treatment of medical and surgical cases will be given by Dr. French, physician, and Mr. R. P. Rowlands, surgeon, Guy's Hospital, on Wednesday evenings, commencing February 27, and ending on April 3, in the Nurses' Home at 8.15 p.m. The fee for the course for members will be 6s., and for non-members, 7s. 6d. The lectures will be printed in pamphlet form and will be sent out to subscribers in due course. Intending subscribers should send in their names to the matron as early as possible.

### RESIGNATION OF A LONDON HOSPITAL MATRON.

The post of matron of the London Homœopathic Hospital has become vacant by the resignation of Miss Victoria Daunt, who succeeded Miss M. Brew in November 1905. Miss Daunt was trained at the Great Northern Central Hospital, where she was afterwards sister for several years. She subsequently went to the National Hospital for the Paralysed and Epileptic as night sister, and was for a short time acting matron. The salary attached to the vacant appointment is £100 a year; and candidates must be over 30 and under 45 years of age. Applications should be sent to the Secretary of the hospital, Great Ormond Street, Bloomsbury, W.C., by March 1.

### EAST LONDON HOSPITAL FOR CHILDREN.

At the half-yearly Court of the East London Hospital for Children on Monday last the Board expressed their pleasure that the Lady superintendent, Miss Rowe, who had been absent for three months, was able to return to her duties, and tendered her their most sincere sympathy for the cause of her absence.

### IN THE ABSENCE OF THE NURSE.

An extraordinary incident has taken place at Reigate and Redhill Borough Isolation Hospital. A boy of fourteen, a patient in the diphtheria ward, during the absence of the nurse, jumped through a window, and, scaling a high gate, got away. It appears that he then walked through the mud for two miles, obtained access to his father's house without rousing the family, and was found the next morning by a Redhill police officer partaking of tea and toast in the kitchen. When it was discovered that the boy was missing, the matron and nurses at once made a search for him, and were unable to find him. He has since been taken back to the institution. It is difficult to say without knowing the manner in which the hospital is worked where the fault lay, but we are quite certain that it ought not to be possible for a patient to leave it unperceived in the middle of the night.

### A MANCHESTER NURSE AND THE JAMAICA EARTHQUAKE.

We hear of another nurse who distinguished herself in her efforts to succour the injured at the time of the earthquake in Jamaica. One of the latter was Sergeant Sharpe, of the Royal Army Medical Corps, and his wife, the daughter of Staff-Sergeant Bright, of the Manchester Medical Volunteers, rendered the most valuable assistance. After she had assisted to patch up her husband, who was badly hurt, she helped in the camp as long as help was required, and then continued her work day and night among the married people. She herself, with her little baby, had a narrow escape. Before her marriage, three years ago, Mrs. Sharpe was a sister at West Didsbury Ho[illegible]al, where she was trained.

### A LECTURE AT GUY'S NURSES' HOME.

In connection with Guy's Hospital Photographic Society, which is a branch of the Nurses' Recreation Society, an interesting meeting was held in the Nurses' Home on Thursday evening last week. Miss M. Smith, Secretary of the Society, gave a lantern lecture entitled "A Ramble through London with Charles Dickens." The slides were excellent, and some of them had been taken and prepared expressly for this lecture by Miss Smith. As many of the well-known haunts appeared in view the lecturer conducted her party in imagination from the familiar Borough and over London Bridge to St. Paul's, then on to Holborn and Staple's Inn, and finally back to "Guy's entrance gates." The references to Mrs. Gamp provoked ludicrous comparisons between the fitness of the past and present nurse for her profession. Buckingham Street, too, with its present suggestion of massage, came in for some amount of interest in association with "Miss Betsy Trotwood," who once had apartments in a house still standing there. The proceeds from the tickets go to supply needed funds for the prizes for the annual exhibition of photography at Guy's.

### A NURSE AS SUFFRAGETTE.

Miss Olivia Smith, one of the fifty-two "Suffragettes" arrested last week for making a disturbance outside the Houses of Parliament, describes herself as a nurse of Clement's Inn, and wore a nurse's costume in court. The evidence against her was that she "deliberately got hold of the bridle of a mounted officer's horse and shoved it about." Her explanation was that she "had already shoved one horse off the footpath, and the policeman said, 'There's another there; go and shove that.' Not liking to break the law, she went and did as she was told." The magistrate ordered her to pay forty shillings or go to prison for a month; and as she elected to go to gaol, she was sent to Holloway.

### CONFERENCE AT ALNWICK CASTLE.

The Duchess of Northumberland entertained at Alnwick Castle a few days ago a large number of delegates representing the various districts working in connection with the Northumberland County Nursing Association. This Association numbers 72 nurses and two relief nurses. Mention was made in the tenth annual report, which was discussed at a conference in the Great Hall of the Castle, of the death, during the year, of Lady Grey, who took the keenest interest in all kinds of nursing.

### SHORT ITEMS.

The late Lord Grimthorpe has left £1,000 to "the Victoria Nurses' Fund" and £100 to "the Hon. and Rev. R. Grimston's Nursing Fund."—Miss E. H. Beecher, R.R.C., Principal Matron in Queen Alexandra's Imperial Military Nursing Service, has been appointed secretary to the Nursing Board in succession to Lieut.-Col. B. M. Skinner.—On Wednesday evening last week Miss Studley, Principal of Long's Swedish Gymnasium and Institute for Medical Gymnastics and Massage, gave a lecture to the members of the Irish Nurses' Association, and with the help of some of her assistants practically demonstrated some of the movements used in the treatment of various cases.

### The Nursing Outlook.

"From magnanimity, all fears above;
From nobler recompense, above applause,
Which owes to man's short outlook all its charm."

### MAKE-BELIEVE IN BRITISH NURSING.

### II. A COMEDY TO BE ENDED OR MENDED.

We showed last week from the report of the Matrons' Council that, for practical purposes, the National Council of Nurses, the Society for the State Registration of Trained Nurses, the British section of, and indeed the International Council of Nurses also, as will appear directly, depend for their bone and sinew on some eight or ten ladies, several of whom are not now actively engaged in nursing, but who call themselves the Matrons' Council of Great Britain and Ireland. How seriously some of these ladies take themselves is well illustrated by their official announcement, that two of them, "intend to visit Paris *after the opening of Parliament*." Another humorous touch is the method pursued to make much out of little, by the use of high sounding titles to cover the weakness of the position, due to the smallness of the Stage Army, and to the absence of the names of the leaders of nursing in this country. Thus, the Stage Army managers, in order to remove "some misapprehension concerning the organisation of the Conference in Paris," "make it plain that all such organisation is in the hands of the officers of the International Council of Nurses—Mrs. Bedford Fenwick, founder and honorary president; Miss McGahey, president; Miss L. L. Dock, honorary secretary; and Miss M. Breay, treasurer; together with the Councillors, the British and American Matrons who are Foundation Members."

Here it will be noticed that the third International Conference, after all these years, is still to derive its bone and sinew from the Matron's Council, the constitution of which was set forth in our article last week. Both Miss McGahey, the late lady superintendent of the Royal Prince Alfred Hospital, Sydney, who was trained at the London Hospital, where she was at one time a matron's assistant, and Miss L. L. Dock from the United States, whose ability no one will question, are honorary members of the Matrons' Council. We cannot ourselves believe, that, either Miss McGahey or Miss L. L. Dock, in fact, fully realise that, in associating themselves with the Stage Army, they are placing themselves in opposition to the sentiments and opinion of the accredited leaders of British nursing, and to the views and wishes of the great body of trained nurses in Great Britain. However, if they are fully conscious of these facts, and are acting with deliberation, it is well that they should be reminded that, the non-representative character of the Matrons' Council, and all that appertains to it, must react upon the International Nurses' Council. If the latter Council is to fulfil any useful purpose, it must be made a representative body, and must include the accredited leaders of nursing in every country, which is claimed to be represented at each Conference it may hold.

It would, then, be a calamity should foreign countries not fully realise that, at present, British nursing is not represented by its responsible leaders, or in any real sense at all, on this so-called International Council of Nurses. These leaders have, hitherto, been excluded from it by circumstances, which are perfectly well known throughout the nursing world. There is, therefore, in reality, no representative International Council of Nurses. For this reason, mainly, the so-called International Congress has been regarded in this country as a comedy. This view is based on the facts we have published in this and a former article. No one questions the ability of some, at any rate, of the ambitious women who are responsible for the comedy in question. It is to be regretted that they should not have used their brains to better purpose, and, by sinking all personal ambition, have brought themselves into line with the best nursing opinion, by putting an end to the Stage Army and its proceedings.

We have felt it to be our duty, as the most representative and oldest nursing paper, to call a halt at this stage of the masquerade, and to ask every member of the profession to look the whole matter squarely in the face. It is certainly due to British nursing, that the true inwardness of the proceedings of the organisers, of the proposed International Congress in Paris, should be promptly and clearly brought to the knowledge of the Director of the Assistance Publique. It would be a calamity, and most unfair to this most courteous and able hospital authority, not to put him and all other French authorities in possession of the actual facts.

England has been the foster mother of modern nursing. It is most important that all nursing authorities in foreign countries should clearly understand, that, British nursing is, in no real sense, represented by the small body of matrons and ex-matrons, whose ambition it is, to control nursing affairs in this country, and to largely dominate nursing affairs all the world over, through the so-called International Council of Nurses. If an International Council of Trained Nurses is ever to prove of practical value, or to escape from the perilous risk of becoming the laughing stock of most that is best and intelligent in modern nursing, it is essential that it shall be made representative of every great school of nursing, and also of trained nurses throughout the world. At the present time, the attitude assumed by a few ambitious women, who have pushed themselves forward as reformers, is merely blocking the way of all joint action, of all national courtesy, of all unity of aim. And yet they boast that theirs is a campaign of nursing reform!

### Nursing in Tropical Climates.

By Andrew Duncan, M.D., B.S., M.R.C.P., F.R.C.S., Fellow King's College, Lecturer on Tropical Medicine at the London School of Tropical Medicine, and the Westminster Hospital.

### VII. DYSENTERY.

The nurse will not be long in the tropics before she has a case of dysentery under her charge. Formerly this was held to be one disease; it is now known that under this term are included several varieties "of intestinal flux, the acute forms characterised by pain, frequent passage of blood and mucus, the more chronic by diarrhœa alternating with constipation and a tendency to recurrence. Usually there is inflammation, and in the chronic cases ulceration of the large bowel" (Osler).

### Causation.

Briefly, there are three varieties of the complaint —one caused by a bacterium, one by an amœba, and one the dysentery found in war in which no organism is found, as in our recent war in South Africa. As regards the predisposing causes, we may mention—

### 1. Season.

In the tropics dysentery is less frequent in the cold season, becoming more common in the months of heat and drought, and commonest of all towards the end of the rains and beginning of the dry season—that is to say, in those months corresponding in temperate climates to the end of summer and autumn. The worst time for dysentery is at that period of the year characterised by extreme fluctuation of the temperature from hot days to cold nights. In Bengal it rages chiefly during the rainy season.

As regards the effect of *atmospheric moisture* or of *rain* and *dew*, opinions are somewhat divided, some authorities holding these factors to be highly important, others holding that they are not so. Hirsch inclines to the latter view. He instances 126 epidemics, 65 of which arose with the wet weather, 61 making their appearance in the midst of continuous dry weather and lasting through it.

### 2. Influence of Soil.

Where a soil is organically polluted, and dusty so that infection is conveyed by the wind, dysentery is very likely to be prevalent.

### 3. Malaria.

There would seem to be an indirect connection with malaria—namely, dysentery is very likely to prevail in the vicinity of swamps and marshes in porportion to the intensity and frequency of malarial fever. The mosquito, however, has not yet been held to convey the disease.

### 4. Conditions of the Food.

(*a*) *Impure drinking water.* This has ever been a cause in our campaigns—for example, in the Egyptian War of 1882 much of the dysentery was due to the foul canal water drunk by our men. In our late South African War the Commission on Dysentery and Enteric Fever reported that the most frequent cause of the prevalence of dysentery was probably the drinking of the surface waters, especially those from the rivers which contained a very large amount of mineral and organic matter in suspension. A large amount of disability thus occurred in Lord Roberts' march to Bloemfontein. A third example may be cited from the medical history of two French regiments of the line. During August of a certain year the 19th and 44th Regiments were barracked at Neuilly. The water was drawn for both regiments from the same source. The 19th regiment drank their water with brandy, the spirit of which precipitated the organic matter contained in it, and this putrefied in the contained vessel. The regiment was attacked with severe dysentery. The 44th Regiment used their water in making tea and coffee, the tannin of which prevented putrefaction. They only suffered from slight diarrhœa. Red wine was substituted for the brandy in the 19th, when the dysentery ceased. Where the disease results from impure water amongst large bodies of men, many patients will come into hospital.

(*b*) *Intoxicating liquors.* Intemperance greatly predisposes to the disease. In war after the capture of a city, unless strict precautions are taken by placing sentries over the wine-shops, much dysentery will ensue.

(*c*) *A monotonous diet* will favour the occurrence.

(*d*) *Salt rations.* In the first Burmese War (1824-26) for six and a half months the troops were fed on salt rations, and 48 per cent. perished principally from dysentery. Here the cattle were, in the first place, marched to Calcutta from distant stations and slaughtered in February 1824 under a degree of heat so great that decomposition must have set in. The flesh was then salted. Again, in the China War of 1840, notwithstanding this terrible precedent, we find the Government had learnt nothing. Cattle were again marched to Calcutta, and slaughtered in the heat of February, with the same consequences to the British troops. The meat was half putrid when the force sailed. In one regiment (the 26th Cameronians), embarking 900 strong and full of health, the result was that at the end of two months there were not 200 men left fit for duty in the field, owing to the havoc made by scorbutic dysentery. But even where the salt ration is not decomposed before salting, we have two evil factors: first, the irritant effects of a salt diet; secondly, the insufficient nutritive power, whilst the salt ration of military life is more highly salted than usual in order that it may keep for two or three years in any climate.

(*d*) *Excessive diet of fresh meat.* Often after raids in Algeria the French soldiers have been seized with this complaint from eating largely of the fresh mutton from the captured sheep.

(*e*) *Tinned provisions.* These, if largely used, as instanced in the Nile Expedition of 1884-86 and in the South African War, will bring on the affection.

(*f*) *Imperfect preparation of grain food.* In Lord Roberts' great Cabul-Candahar march the native followers often arrived in camp too tired to cook their food properly, and so ate uncooked atta and Indian corn. Dysentery followed.

(*g*) *Fruit.* Trousseau held that fruits even when not ripe could not cause the disease. Horton dissents from this. In my experience in Afghanistan fruits played but a small share in the causation of dysentery. An almost unlimited supply of grapes and melons was at hand in the Kurram and Logar Valleys, and I never traced any of the dysentery to this supply.

(*h*) *The influence of flies.* Of all living things the fly is probably the most obnoxious. And those only who have been in the tropics know what an obnoxious plague the fly is. In the South African War the myriads of flies in the circumscribed camps were one of the chief means of spreading dysentery and enteric fever. Infesting the latrines, they carry the infecting material not only on their heads, but also on their bodies, legs, and wings.

(*i*) *Long occupation of the same ground by any large body of men.* In war any camp site should, if the military exigencies of the situation permit of it, be changed at intervals, otherwise the soil will become polluted with the excreta of men and animals, and bowel affections will inevitably arise. Morache draws especial attention to the effects produced by not changing camp at regular periods. The presence of a large number of men persistently remaining on one spot must inevitably infect the soil, whatever precautions are taken; organic matters from excreta of men and animals are trampled into the earth; foul water from various sources, such as from the kitchen, etc., is also imbibed from the soil, and soon a veritable forcing-bed for zymotic diseases is formed. And how this process must be advanced under a tropical sun! The infection of the soil thus arising is of itself sufficient to start the various forms of bowel complaint, including epidemic dysentery. A good example of this occurred in the New Zealand War in 1864, where, after the occupation of Waikato, several Government officials were employed in surveying the district. They constantly changed their camping-ground and remained perfectly healthy. After a time for protection they were obliged to pitch their camp near the troops, and kept on the same spot. Bowel complaints soon broke out, the more so as their camp was near that of the Land Transport Corps, who had remained encamped on the same spot for some time, and whose camp itself was notoriously unhealthy. As soon as they were able to do so, the survey party changed their site, and the bowel complaints were at once arrested.

5. NEGLECT OF PERSONAL PRECAUTIONS.

In the tropics, especially on service, a neglect of personal precautions is a fertile source of dysentery. Thus, one should never sleep in immediate contact with the ground. One should always wear the cummerbund at night. Especially is it necessary to sleep under cover where the dew is drenching and heavy. A good example of this was shown in the Egyptian War of 1882, where the Manchester Regiment, under good cover at Ismalia, escaped the drenching dews that the rest of the force not located there were exposed to, and scarcely suffered at all from the bowel complaints which were so rife in the latter.

6. NEWCOMERS IN THE TROPICS

are especially predisposed to bowel complaints, due doubtless to the relaxing effect of the heat on the system. In a previous lecture allusion has been made to the opinions of Laveran and Brydon on this point, so that no more need be said on the subject beyond this—that the nurse should, on her arrival in the tropics, take all the necessary precautions (to be detailed presently) against these affections.

Two examples will fitly conclude this section of the causation of dysentery. In the Ashanti Expedition of 1863-64 great aggravation of the dysentery returns became evident. Out of 210 cases in 1864, 163 occurred in the rainy months—April to July—and out of 32 deaths 26 also occurred at this period. The huts leaked at the Prahsu Camp, rains having set in before the latter was finished. There were vast forests, a river, and dense jungle composing the site of the camp, which gradually became a swamp. The days were very hot, the nights very cool, and the food of salt meat, biscuits, and bad water. And, again, in the Malay War of 1875-76 dysentery truly scourged our men. In the Buffs the admission rate was 70.11 per 1,000. But two companies of the latter were kept stationary at Malacca; they had no mortality and great immunity from the disease. Why? On account of the light duty, good quarters, no sun exposure, good food, and midday heat tempered by a mild sea breeze: the mortality was 8.34 per 1,000 in the rest of the regiment located elsewhere.

In the next lecture the signs and treatment of dysentery will be dealt with.

(*To be continued.*)

### The Nurses' Clinic.

### GASTRIC CASES.

MANY and varied are the kinds of gastric diseases nurses must meet with in their daily work. It is a proud hour for the probationer when she gives her first feed to a case of gastrostomy, under the superintendence of the head nurse, who impresses upon the novice the importance of carefully mixing and straining the liquid diet. All gastric cases call for great skill and patience from the nurse in charge. Take, for instance, a case of gastric ulcer. A patient may have suffered from this complaint for years in a chronic form, or may suddenly develop it in an acute form. The pain is, perhaps, mistaken for dyspepsia, and the patient keeps about until an attack of hæmatemesis occurs, and a doctor and nurse are sent for. If the nurse arrives first, she must see that the patient is kept lying absolutely flat, and not on any account allowed to sit up or make any movement unaided, all talking and excitement to be forbidden. The foot of the bed should be raised, fresh air admitted, and the limbs protected from cold by hot-water bottles. On no account let the patient take anything by mouth (particularly stimulants) till the

**THE NURSES' CLINIC.—*Continued.***

doctor has seen the case and given his orders; he may allow ice to suck in small quantities, it allays thirst, but is provocative of wind and not always allowed.

Most probably blood will be passed in the stools, giving them a black, tarry appearance; this must always be watched for and noted.

The treatment varies slightly, according to the physician, but many prefer to give the stomach entire rest by employing rectal feeding for some days at least. After that, food by mouth is begun in very small quantities, ℥ss. peptonised milk half-hourly till larger feeds at longer intervals can safely be taken. Afterwards plain milk may be tried, then a little clear soup, then gradually return to light farinaceous, and if no pain is felt a little boiled fish may be added, and finally chicken. The slightest return of pain is an indication that food must not be pushed, and it is certainly very disheartening to find that time after time a patient has to return to peptonised milk, but one must walk before one can run.

Should a gastric ulcer perforate, the outlook is very serious. An operation will probably be performed to sew up the hole, and the after-nursing is like any other severe abdominal section, great care being taken to prevent collapse, and to be on the watch for peritonitis.

Gastralgia is a very trying complaint, which can only be successfully treated by prolonged rest and strict attention to diet and such medicines as may be ordered. The patient complains of a burning feeling in the stomach, and almost any food aggravates the discomfort, the appetite is naturally very poor, and the patient so dreads eating that his condition is rapidly enfeebled.

The great thing on the nurse's part is always to prepare the food in as tempting a manner as possible, and not to serve up more than can safely be taken at one meal. It is never too much trouble to try recipe after recipe if, at last, something is found which the patient can take with a minimum of discomfort. I knew a case where, after having tried everything, a diet of pounded yolks of hard-boiled eggs was discovered. For some time the patient lived upon nothing else, and from thence gradually made a return to light food, and finally recovered.

Gastritis, or inflammation of the stomach, is another complaint which needs skill in nursing. The patient is sometimes almost doubled up with pain, and constantly sick after food. Warmth and rest in bed and the lightest, most digestible food in small quantities, must be given. Benger's food, peptonised beef-tea, essence of chicken can be taken, but in cases of this kind the diet will be ordered by the doctor.

Cancer of the stomach is often met with in hospitals and districts. Probably the patient is a working-man, who has suffered for years from "indigestion" and taken no notice of it till increasing sickness and emaciation force him to seek advice. He is very hopeful that a course of medicines will put him all right, but unless the disease is so far advanced as to be inoperable, an operation is almost a necessity. The patient must be prepared as for any other major operation, and warmly clad in flannel jacket and woollen stockings; hypodermic stimulants are, of course, to hand, as sometimes the shock is very great. After the operation he is put back into a well-warmed bed with a hot blanket next him, a cradle over the abdomen, a pillow under the knees to prevent any straining on the operated area, and he must not be left till he is well round. Nothing at all to be given by mouth at first, but the mouth may be frequently rinsed to clear it of the sickly taste of anæsthetic and to relieve thirst. A pint of tepid water given very slowly per rectum will also allay thirst, and is often ordered. As in all abdominal operations, flatulence is very trying for some hours after, the long rectal tube carefully oiled or smeared with vaseline and gently passed upwards and backwards into the rectum will disperse the wind and relieve the pain. The other end of the tube should rest in a dish.

Gastro-enteritis is very common amongst young children, particularly in large towns. Improper feeding, chills and insanitary conditions are pre-disposing causes, and as prevention is better than cure, a nurse can do much by urging the importance of good, boiled milk, simple wholesome food, warm underwear and cleanliness. Mothers often think they have done their duty after administering a dose of castor-oil, that favourite panacea for all ills amongst the poor, but gastro-enteritis is the cause of a large amount of infant mortality, and can often be prevented by a little common sense and forethought.

### Incidents in a Nurse's Life.

### DISINFECTING WORKHOUSE PATIENTS.

I was in charge of the "contact house and out-bathing station" of a town on the south coast during an outbreak of small-pox. We (myself, a maid, and a porter) had been there about four months, and things had quietened down, so that we were expecting every day to be recalled to the hospital to which we all belonged.

I had just finished supper one night and was thinking of bed, when a loud knocking at the front door startled us all. "Another case," I said to myself, for I knew no one but the medical officer of health would knock so late. "A case of small-pox at the workhouse, nurse," said the doctor as soon as he got in. "Eighteen contacts, most of them old and infirm; it is too late to bring them here, you must come with me, and we will disinfect them at the workhouse."

"And the case?" I said.

"Oh, it is too late to send him to 'the ship' to-night; he must come here, he is not so very bad, and you can have him transferred to 'the ship' as soon as the tide is up in the morning."

When we arrived at the workhouse I found there were two male nurses—they were waiters on a yacht during the summer—who were to bathe the men; while a very stout monthly nurse (who had been fetched over from the female block and was very irate about it) and myself were to put the old men's heads in hyd. per. chlor. caps and superintend generally. But before the doctor would allow us to go into the ward I had to cover my uniform with a patient's nightshirt with my apron on the top, remove my cap, and substitute a small towel, which left no hair visible, while the nurse was arrayed in an elaborate nightdress belonging to the matron and her head covered in similar fashion. Attired in this way we entered the ward. I wonder the patients did not think that they were delirious when they saw us coming. I had never seen a workhouse ward before, and my first feeling was one of thankfulness that I had only come to disinfect and was not expected to stay there.

There were about eighteen beds, in each was an old man; every patient able to get up in the day had his clothes spread over his bed; there were dirty glasses, cups and saucers, and half-empty bottles of soda-water standing about on lockers; while, crowning horror, the place simply swarmed with bugs, and the patients' pillows were stained all over where they had smashed them.

Nurse and I set to work to get the poor old men ready for the male nurses. Many of the poor old daddies were thoroughly frightened at the unusual fuss and also cowed at the prospect of a bath which was evidently a luxury they were not used to. I heard our doctor ask the master how often the men were bathed, and he said, "They have a bath when they come in, and the Lord knows when they have one after," and some of them had been in for months! However, when the doctor ordered them each to have a dose of hot whisky and water after their bath there was not one who would not have been willing to have been bathed ten times over!

The bath-room led out of the ward and was about as clean as the rest of the building. The floor consisted mostly of a trap-door, through which the contacts after bathing were taken down a ladder to a non-infected ward. When this door was open there was so little room that we had to sit on the edge of the bath. We were too busy to think of it then, but it must have been an absurd sight, nurse, myself, the doctor, and the master all sitting in a row on the edge like so many little birds on a bough, while the male nurses took the contacts down.

While we were busy disinfecting, the matron's beautiful white Persian cat strolled into the ward, and doctor insisted on it being bathed in perchloride, from which it emerged pale blue, much to the indignation of his mistress. We got through with them all at last, and at 1 A.M. I arrived back at the contact house, ready to do night duty with the small-pox patient who had preceded me and had been put to bed by the porter in my absence.

This was my first and last experience of workhouse wards; and I may add that the workhouse referred to has now disappeared, and a thoroughly up-to-date building, under proper management, has taken its place.

### An Opening for Preliminary Training.

### INTERVIEW WITH THE LADY SUPERINTENDENT OF THE ALEXANDRA HOSPITAL FOR CHILDREN WITH HIP DISEASE. BY OUR COMMISSIONER.

The mere fact that her Majesty the Queen is patron of the Alexandra Hospital for Children with Hip Disease and takes a great personal interest in its welfare would suffice to invest it with interest. But the kind of knowledge which is gained by the probationers engaged in this admirable institution is hardly so well known as it might be. On the occasion of my visit to the hospital, the lady superintendent, Miss E. M. Fitch, told me that a change of some importance has lately been made in the regulations for the admission of probationers.

### The New Rule.

"For the future, candidates between the ages of 18 and 27 will be taken for one year's course, but they will receive no salary, and will provide their own uniform and laundry expenses. If they enter for two years, they will receive £12 the first year and £13 the second, in addition to uniform and laundry; but candidates for two years' training who are under 20 do not receive any salary the first year."

"Then you take them at 18?"

"Yes, this institution expressly offers an opening to girls under 20, who want to get into a hospital before they can obtain general training. We do not give certificates, but there are lectures by members of the medical staff on surgical work and general nursing, starting in October and continuing to March. An examination is held at the end of the course. At the end of two years our probationers leave with a good idea of anatomy and with valuable experience in ward work.

### The Ward Work.

"I see that the children are tied down in their cots?"

"Without exception, owing to the nature of the disease. Every child—there are 68 in the hospital just now—is particularly helpless and has to be washed and dressed. The ages run from three to 12, but the average is between six and seven. The patients being so young, there is no heavy lifting. That obviously makes a difference in the nursing. But the general care of the children is no slight matter, and some of the cases are also bad."

"What are the worst kind of cases?"

"Those of meningitis, but they are very uncommon. We often have cases with many abscesses, and there are a great many wounds, so that there are plenty of dressings to be done, and as there is no house surgeon the probationers have to help the sisters with them."

"Have you any cases of bed-sores?"

"None at all! We have most beautiful mattresses, specially made and very thickly stabbed, so as to be very flat. The cots are high, so that there is no work involving backache. The probationers have night duty for three months at a time; they go into the wards at 8 P.M. and leave at 8 A.M., are off duty until 12 noon, and have a night off once a month. There are two probationers on night duty and a sister in charge of the wards. The work at night is usually light, because, except in a certain percentage of patients who wake up and want drinks, the children sleep for hours."

### The Day Duty.

"I consider," continued the matron, "that the wards are well arranged for nursing. As you observed in going round, there is a kitchen in the middle of the two wards connecting them on each floor, the wards themselves holding thirty-four cots. Some of the cases are taken out to the balcony which was only finished in 1905. They all do extremely well outside."

"How is each floor staffed during the day?"

"With a sister, a staff nurse who is trained, and four probationers, there being also one probationer available as a relief nurse. The staff nurses and probationers are on duty at 7 A.M., and have supper at 8.15, but they have two or three hours off duty every day, half a day every week, and a day off once a month; also theatre leave once a month till 11.30. The holidays are sixteen days the first year and twenty-three days the second; if possible, I like the probationers to have a holiday when they have been here six months, because it is so important that they should not get fagged out. But there is no rush of cases here, and the work altogether is very level. The nurses, too, are well looked after and have to spend an hour out of doors every day."

### The Question of Diet.

"Is outdoor uniform compulsory?"

"No, there is a recognised outdoor uniform, but I do not think it is necessary to wear it. Those who wish to wear it get it for themselves and a dress to match, as the cotton dresses may not be worn outdoors. No one is allowed to wear it who has not signed for one or two years."

**AN OPENING FOR PRELIMINARY TRAINING**—*continued.*

"Then you have a preliminary period?"

"Yes, all probationers have to come for two months, which are not included in the two years' training; at the end of that time I generally know if a girl is suitable, but she has to be tested for physical fitness by our own physician. This is a very important examination, because of the immature age of some of the probationers."

"Your accommodation for the nursing staff is entirely in the hospital?"

"The hospital was built with that view. The accommodation consists of a very fair dining-room, a comfortable sitting-room with piano, and bedrooms containing two beds. But, as you can judge for yourself, they are large and airy. I lay stress upon the importance of diet. It is too bad to give nurses mutton all the year round, and they have as much variety as possible. The same applies to the patients. For instance, on Sunday the children had cold mutton and milk pudding; on Monday hot roast mutton and milk pudding; on Tuesday fish and bread and butter pudding; on Wednesday steak pudding, custard and prunes; on Thursday lentil soup and treacle roly-poly; on Friday roast beef and milk pudding; and on Saturday soup and jam roly-poly."

An Out-Patient Nurse.

"How large is your staff?"

"There are an assistant-matron, three sisters, two staff nurses, and twelve probationers. The duties of the sisters are much the same as those in ordinary hospitals. A staff nurse takes a sister's place when she is off duty. Then we have an out-patient nurse. She hunts up the out-patients at their homes and sees that they come to show themselves to the surgeon when it is necessary. I am sure she does a great deal of good. If she finds that the children are not doing well they come back here or they go into one of our homes. These homes are at Clandon, near Guildford, and at Painswick. There are twenty beds at the former, and the staff consists of a matron in charge, a night nurse, and three day nurses. At Painswick they are supposed to be convalescent children, and the matron in charge has two nurses for the twelve cots."

"You have not been a matron a very long time?"

"Two years."

"Matron, is the Queen Coming?"

"I suppose there has been a considerable development of this institution?"

"You mean since the Queen, then Princess of Wales, opened this building in 1899. Before that time nurses had to pay to come here. In the old building there were no wardmaids; the ladies had to scrub the floors. The Queen's interest in the hospital dates from 1881, and also that of Queen Maud, who came here as a small child. We have still some of the Danish toys brought by her Majesty four years ago. She sent sweets on her last birthday, and a small boy, when he heard the news, said, 'Matron, is the Queen coming?' The children are always hoping that she will come again. We have a good many presents sent us, but the clothing of the patients is always a difficulty. They wear things out so quickly. I can always find patterns and sometimes material if kind people will only make the articles."

"Do your probationers on leaving the hospital experience any difficulty in obtaining training elsewhere?"

"I do not think so. I get a great number of applications, but I am very careful to secure, if possible, those who, in the ordinary course of events, should be able on leaving here either to take children's training or, after the necessary age interval, to enter a general hospital."

### Strapping a Leg for the Cure of an Ulcer.

### EXAMINATION QUESTIONS FOR NURSES.

The question was as follows: "If ordered to strap a leg for the cure of an ulcer, how should you proceed? Describe your preparations, mode of procedure, and finally in what manner should you remove the strapping when necessary."

First Prize.

The limb should be cleansed, the area to be strapped shaved, the soap well washed off and the limb thoroughly dried. Then prepare the materials required: Strapping cut into strips not more than one and a half inches in width and about eighteen inches in length, a strapping can or jug filled with boiling water, a bandage, and scissors.

In the case of a brawny callous ulcer the doctor will probably order the whole of the part to be covered in, but in the case of a discharging wound, "a window" to allow of the escape of the secretion and the application of a dressing, should be made, as the strapping will to a certain extent absorb the discharge and irritate the tender edges of the wound.

To strap the whole of the part: The heel should be raised on a support and the nurse stand facing the patient. The strip should be thoroughly heated by holding the plain side against the strapping can, and the centre applied to the back of the limb about two and a half inches below the ulcer, the ends brought over the sides, crossed in front (the inner end crossing the outer) in an upward direction and carried round toward the back. The strapping should fit the limb perfectly without any snipping. The next strip should be applied in the same manner but should overlap the first for about a third of its width, and so on until the limb is covered for about two and a half inches above the wound, where it should be neatly finished off by a strap applied horizontally. Care must be taken that the strapping be applied with even pressure, and firmly enough to keep the part at rest, but not so firmly as to cause discomfort to the patient. An ordinary bandage should be applied over the strapping.

To strap an ulcer leaving a window: In addition to the materials mentioned before, some shorter pieces of strapping will be required and the dressing ordered for the ulcer. Proceed to strap in the same manner three inches below, and cover to the lower edges of the wound. Then apply the shorter pieces of strapping to the sound part of the limb, leaving the ulcer exposed. The strapping should be cut to fit exactly to the edges of the wound. Above the ulcer the strapping is carried right round the limb again and finished off neatly. The raw surface should be covered with a swab and not left exposed to the air while the limb is being strapped. Then apply the dressing to the ulcer. Boracic ointment is usually ordered, though in some cases good results have been obtained from lint soaked in rubra lotion or picric acid one per cent. Whatever dressing is applied should fit inside the strapping and not overlap at all. Next apply protective and bandage.

To remove strapping. Pass a director under the strapping at the back of the limb and cut with scissors. The strapping can then be removed in one piece, freeing it first from each side, pressing down the skin with one hand while doing so and drawing it toward the centre. It must be very gently detached from the ulcerated part so as not to injure any newly formed tissue. The marks remaining can easily be removed by rubbing with a little olive oil or vaseline (these are less irritating to the tender skin than turpentine, which is usually recommended), and washing with soap and water.

"Challie."

Second Prize.

To strap an ulcer I would first prepare the strapping, cutting the required lengths an inch wide and long enough to go round the leg and cross in front. Have ready also some boiling water. Then I would prepare the patient by first thoroughly washing the leg with soap and water and if necessary shaving it, swab the ulcer with antiseptic lotion, and apply a compress the size of the wound, which is removed when nearing the wound with the strapping. Place the leg on a support. If no directions have been given as to the extent of the strapping, commence three or four inches below the ulcer and continue for the same distance above. Pass the non-adhesive side of the strapping round a jug containing boiling water, then fix by its middle to the back of the leg, bring the ends firmly but not too tightly forwards, and cross on the outer side of the leg unless the ulcer is on that side, when the crosses must be made on the inner side, avoiding the prominence of bone. Each succeeding strip must overlap its predecessor by about one-third its width. Very often the ulcer is left exposed so that the dressing can be frequently renewed. The strapping must then be cut neatly around the edges of the wound.

Keeping the crosses even down the side of the leg and avoiding exposing the wound to the air more than absolutely necessary. To remove the strapping, I would first press my finger on the flesh below the strapping, again avoiding a prominence of bone or the site of the ulcer, then slip a director, with its grooved surface upwards, in the indenture made by the finger under the strapping and cut upwards with sharp-pointed scissors, taking care to keep in the groove of the director. Draw the strapping off by both sides in the direction of the wound, to prevent the risk of tearing or breaking down of any union which may have taken place, placing the hand firmly on the patient's skin underneath the strapping when drawing it off, otherwise it is apt to pull the tiny hairs on the skin and cause unnecessary pain. Strapping marks may be removed by oil, and the leg then thoroughly washed again with soap and water.

"DAFFODIL."

### THE PRIZE WINNERS.

"Challie" gains the first prize, for she has considered the subject all round. She speaks of the necessity of shaving the leg—this is most important, and the nurse who neglects to do it shows herself a clumsy performer. There is always pain in taking off strapping even if the limb is not particularly hairy, because everyone's legs have more or less fine and almost invisible hairs, but they are enough to cause acute momentary suffering when strapping is taken off. "Challie" also has the right method in removing it, as she says, in one piece, as it is a fatal error to pull off each piece separately, as the larger number of candidates suggest. It is quite right to cut up strapping at the back and pull from both sides towards the ulcer, but this should be done as gently as possible, though firmly, quickly, and without hesitation. "Challie's" window, too, is well arranged, if ordered, but if permitted to do so, I think it is better to enclose the ulcer with the strapping, leaving some perforations for the escape of discharge. If the patient gets up, it is necessary to strap from the toes to avoid swelling.

"Daffodil," who takes the second prize, sends a good and careful paper.

### HONOURABLE MENTION.

This is gained by "Pretoria," "X. C. D.," "Yorkshire," "Solitary," and "Emesis." "Pretoria's" paper is very good; "Yorkshire," "X. C. D." and "Solitary," though their answers are otherwise good, all fail in taking off the strapping properly.

THE EXAMINER.

### QUESTION FOR FEBRUARY.

1. How would you undress a man who had fractured his left leg?

2. How would you undress a man who had fractured his right arm?

N.B. These two injuries are not supposed to be both on the same patient.

### RULES.

The competition is open to all. Answers must not exceed 500 words, and must be written on one side of the paper only, without divisions, head lines, or marginal notes. The pseudonym, as well as the proper name and address, must be written on the *same* paper, and *not on a separate sheet*. Papers may be sent in for 15 days only from the day of the publication of the question. All illustrations strictly prohibited. Failure to comply with these rules will disqualify the candidate for competition. Prizes will be awarded for the best two answers. Papers to be sent to "The Editor," with "Examination" written on the left-hand corner of the envelope.

In addition to two prizes, honourable mention cards will be awarded to those who have sent in exceptionally good papers.

N.B.—The decision of the Examiner is final, and no correspondence on the subject can be entertained.

Any competitor having gained three prizes within the current year shall be disqualified from taking another until 12 months shall have expired since the first prize was gained.

### Clapham Maternity Hospital.

THE open examination of the Clapham School of Midwifery, which is held three times yearly, took place on Saturday last, and thirteen candidates presented themselves for the usual threefold examination—namely, written, *viva voce*, and clinical.

Of these, two passed "with honour"—i.e., with above fifty marks, sixty being the maximum obtainable—seven "passed," and four failed, the failures being those who did not obtain full two-thirds of the maximum marks. The following are the names of those who obtained certificates :—

Miss Caroline Green (Madras), Miss Catherine Bone, Miss M. R. Parsons, Mrs. Winifred Hanson, Miss Ellen Beadon, Miss M. Scoresby Jackson, Miss Elize Therkilsen (Denmark), Miss A. B. Northcote, and Miss C. Watney.

The questions of the written examination are appended :—

1. What are the causes of retention of urine during and after labour? Describe in detail how you would pass a catheter, and what precautions you would take.

2. What do you mean by tonic contraction of the uterus? How would you distinguish it from uterine inertia? What is the difference in the treatment which is called for?

3. What are the points to be noticed in making

(*a*) A vaginal examination;

(*b*) An abdominal examination in a woman who is in the first stage of labour?

4. What do you mean by uterine inertia? What are its varieties, and what are the causes which give rise to them?

5. How would you diagnose abscess of the breast? How is it caused and prevented?

6. What is meant by post-partum hæmorrhage? How should it be prevented, and how should you treat it?

### International Conference on the Prevention of Infantile Mortality.

THE second International Congress of Gouttes de Lait will be held in Brussels from September 12 to 14, under the patronage of Prince and Princess Albert of Belgium, and many well-known people are taking the greatest interest in the work, which is threefold. First, to give advice; secondly, to encourage breast-feeding; and, thirdly, to distribute milk to those infants to whom breast-feeding is either impossible or insufficient. The Congress has already secured a large membership on the Continent, and the committee hope that England will be well represented. Correspondence on the subject should be addressed in London to Dr. G. F. McCleary, Medical Officer of Health, Town Hall, Hampstead, N.W.

### London Biblewomen and Nurses' Mission.

THE JUBILEE.

The second public meeting to commemorate the Jubilee of the London Biblewomen and Nurses' Mission was held on Tuesday last at Caxton Hall, Westminster, when a very large gathering assembled. The chair was taken by the Earl of Harrowby, and the proceedings commenced by the singing of Schubert's "The Lord is my Shepherd," and "Thou Whose Almighty Word," followed by prayer.

Lord Harrowby then addressed the meeting. He said that the work of the mission had up to the present been carried on in an unobtrusive manner by a committee of enthusiasts, and it was now felt that the time had come to make a general appeal, as it was necessary to get new members and helpers to replace many of the original. They wished to raise a jubilee fund of £10,000, half of which should be devoted to the General Fund, and half to the building of a new Hostel for the workers and the endowment of the Convalescent Home at the seaside. It was also necessary to increase the amount of annual subscriptions. The annual income of the society was £12,000, and the expenses £14,000; though the deficit had till now been covered by legacies. There were eighty-five Biblewomen and seventy nurses employed, and the work of the society was carried on in such a business-like, thorough way that the more investigation one gave to it the more strongly did its needs appeal to one.

The Bishop of London's Tribute.

The Bishop of London, in praising the splendid work of the mission among the very poor, drew a picture of the conditions under which the work is carried out. He said he was competent to tell the meeting of the condition of the dwellers in the East End, as for nine years he had practically lived with them. And the first thing which impressed the worker was the grinding poverty which prevailed. He did not know which was the most pitiable—the man who for weeks tramped London trying in vain to find employment, or the wife who hoped to be able to provide a dinner, or the little ones who begged their mother for bread. Then there was sickness to contend with. He agreed with Sir Frederick Treves, who at the last public meeting of the society had said that it was impossible to deal with all sickness by the hospital. There was much remedial sickness which could have been prevented if attention had been given twelve months earlier. There was also much wastage of life and health due to the fact that patients were discharged from the hospital, and the home conditions nullified the benefits of treatment. It was in encouraging the patients at home and tactfully advising them that the society's nurses did so much to help them. And with regard to the spiritual side of the society's work, the poor needed someone to bear to them a torch of light and to show them what a good woman was. There was a great fog of indifference about spiritual matters, and the poor often resented any suggestion of spiritual help, so that great tact was required in dealing with them. But under this fog he discerned many signs of hope both in respect to the work of the nurses and the Biblewomen, and he was sure that many of those present could tell of instances where they had been the means of bringing health to the poor and of gradually influencing them on the moral side. Those who had actually faced the difficulties were those, indeed, who had the greatest hope for the future. He had not seen much of the working of the society in Bethnal Green, probably because there were already associations for nursing in that district, but he had had testimony to the value of the work they were doing, and read a letter from the Vicar of Shoreditch which spoke very warmly of their assistance. He himself was struck by two or three points in the work of the society. Firstly, they required both the nurses and the Biblewomen to be trained; secondly, they were not a mere dole-giving agency, since no less a sum than £30,000 had during the past fifty years been collected from the poor for the purchase of Bibles. And, lastly, their work was interdenominational—not undenominational—and they all welcomed it as such. Their work was not in vain, and he was very glad to wish them Godspeed.

Canon Walpole stated that he came as witness to the work of the society, and could speak from his own experience in Lambeth of the great good it was accomplishing. He spoke of the heroism displayed by the poor, and added that what they so largely needed was contact with good people. We must multiply our agencies a thousandfold if we wish to really gain London for good; in Apostolic times every Christian was a worker, and we ought to approach nearer to this ideal at the present time.

Professor Clifford Allbutt on the Work.

Professor Allbutt said it was by the happiest accident that he had heard Miss Andrews speak at a friend's house on the work of the society, and he had been so impressed by the value of its efforts that he had determined that, even if owing to his multifarious duties he could not help them in any substantial way, he would do his best to induce others to help them. They worked on the principle that the soul and the body interpenetrated each other, and such a principle, if carried out, as he knew it was carried out by this society, could not fail to have fine results. He said that the human body had passed through long ages of perfectioning until it had become filled with life. Not so the body politic—there were yet large masses unassimilated, full of good and nutrition but unused, and no one knew better the enormous quarry of good value thus unused, owing to the want of good influences, than those who actually worked among the poor. So long as masses remained outside the body politic, they were apt not only to fall into disease, but also to cause disease to the body itself. Some portions had fallen into a kind of atrophy and they needed quickening from the centres of life. The hospitals might, from his point of view, be regarded as the centres of life, and they wanted ramifications from the centres to the large outlying masses; and he could conceive of no system calculated to better achieve this end than the one under which they were working. He drew attention to the fact that a three years' hospital course was to be an essential, they hoped, in future, and when this was so they would have a body of past workers of very long experience and new workers thus thoroughly equipped. He pointed out that in dealing with out-patients at a hospital he had often felt how small a part the medicine prescribed was to play; it was the home life which made for vitality, and what they wanted was the reconstruction of home life. There was the sphere of "after-care," of which the convalescent home was the symbol, and to allow patients to return home without further help and guidance was not only unkind, it was stupid. And there was one thing in the home above all others which militated against health. That was the use of alcohol. He was no fanatic on the matter, but that a fairly decent working man should spend four or five shillings a week out of a wage of twenty shillings was altogether disproportionate. And beyond the care of the body there was the care of the soul. There were these two aspects of therapeutics, and it was impossible for us to be onesided only in our work. It was useless, comparatively speaking, to bring mere material medicine to a man unless one could also bring medicine to the mind. This is what this society recognised, and he felt that the work could be better done by simple Biblewomen than by members of sisterhoods who were apt, he thought, to be the less helpful as nurses because they worked partly for an organisation behind them and often caused a man to be suspicious that a design was being made upon his soul.

The meeting concluded with short speeches from Lord Kinnaird, the Rev. A. Taylor, and the Rev. David Fyffe.

### Everybody's Opinion.

### WHO SHOULD PAY?

"VERNE" writes: If a nurse contracts varicose veins through so much going up and down so many stone stairs during the day from one ward to another, and is told by the chief doctor and matron to have an elastic stocking, should that be provided by the hospital or ought the nurse to provide the cost from her own purse?

### A PROBATIONER LOSES HER SIGHT.

"AN OLD MILDMAY NURSE" writes from Alton, Hants: I beg to enclose postal note for a shilling in aid of the unfortunate probationer who nearly lost her eyesight in the West Ham Infirmary recently.

"POLICY NO. 14203, ROYAL NATIONAL PENSION FUND FOR NURSES," writes: I enclose postal order for two shillings. Can I trouble you to have it forwarded to the probationer who lately met with the accident which has left her unfit for her work? I am very sorry for her. I only wish I could send her a larger sum.

"M. R." writes: I have the deepest sympathy for the probationer nurse who has lost her eyesight through a poor helpless patient suffering from typhoid fever spitting in her eye, and I will gladly send my mite towards anything to help her. But how is it that those who employed her do not compensate, as far as lies in their power, one who is suffering from a calamity caused while she was attending a sick person under their care? Surely the Local Government Board know of the case? [Perhaps the probationer might like to take a thorough course of massage lessons, when she is well enough. That would not be so bad as giving up entirely the work she loves; and if she became efficient she would soon get a good connection.]

### SUICIDE OF A PROBATIONER.

MR. JAS. NASMYTH ROBERTS, The Cottage, Cumberland Road, Hanwell, W., writes: In reference to the inquiry into the death of my niece, Nurse Margaret Kathleen Roberts, reported in your issue of the 9th inst., may I ask you, on behalf of the family of the deceased, to give publicity to their emphatic protest both against the views expressed by the coroner and doctor at the inquest and the verdict of the jury. I was not present at the inquest, but I have been furnished with a fairly full note of the evidence given thereat which to my mind leaves, to say the least, very considerable doubt as to whether Nurse Margaret Roberts took the drug with intent to poison herself. The evidence seems to point the other way. The entire absence of any real trouble, the utter want of reason for the act (the matron's reproof supplies too weak a motive), the nature of the drug taken—strychnine, well known as a tonic and in a bottle not labelled "poison"—taken from a cupboard containing bottles of other better-known and less painful poisons labelled "poison" (or at least let us hope so) together with other drugs non-poisonous in their action, the replacing the bottle and relocking the door, the messages to the matron—sent immediately the drug was taken—to come to her, her earnest appeals to be saved from death, and particularly the absence of any letter or message to any of her family, all negative the intent to kill herself and point directly to the truth of the theory that the poison was either taken as a tonic, or with a view of frightening the matron (her friend), with whom she had quarrelled, and without knowledge of the strength of the preparation she was taking. This view, in the absence of absolute proof to the contrary, is at least as likely a tone as the theory of suicide, in support of which the chief fact was that the girl had a passionate temper, which seems to have, from the very first been openly adopted by the hospital authorities, and followed as a matter of course by everyone else concerned except the unfortunate relatives. It has also the merit of being the most merciful one, and in matters of this kind where there is any doubt it is usual to grant the benefit thereof. It is a matter of regret that the family were not legally represented at the inquiry, as they certainly would have been had they had the slightest idea that the evidence which would be tendered by the hospital authorities would be so markedly one-sided. Had anyone been in attendance on behalf of the family, who could have cross-examined the witnesses and analysed the evidence, the strong probability is that a verdict of misadventure would have been returned. On behalf of the family I am endeavouring to secure an inquiry by the committee of the hospital into the whole of the circumstances surrounding the death with a view of thoroughly sifting what actually did happen in the hopes of vindicating my niece's character, and also as a matter of public duty, for I refuse to believe that in any properly conducted hospital it could be possible for a young and very inexperienced nurse to obtain access to poisons in this way, whether for a proper or an improper purpose. The family desire to be absolutely just to all concerned, and, therefore, pending my application for an inquiry, I will simply confine myself to the above protest, which I trust in all fairness you will be good enough to publish.

### NURSES' EARNINGS, SAVINGS, AND SICK-PAY.

"A GRATEFUL member of the Royal National Pension Fund for Nurses" writes: It appears to me that many nurses would feel much happier if they decided to join the Pension Fund for Nurses and also the sick-pay branch of the Fund. They would then know that they were making a provision for the future, and also that in case of illness they would receive sick-pay sufficient to help them to pay for their medicine and their other incidental expenses, if not to wholly provide for their comfort during the time they are unable to work. It is not easy to save the amount of the monthly, quarterly, or yearly payments out of a nurse's salary, or her fees at a private case, but with a little self-denial it can be done; and when once one has started, it becomes a habit to put aside the amount of the premium first, and to manage with the balance in hand to meet all other demands. As one who has tried, and has also received great benefit from the Fund, and from the sick-pay branch of it, I gladly bear testimony to the help it has been to me. For two or three years I paid in for what would have been sufficient for a fair annuity; then circumstances made it necessary for me to withdraw the whole of the amount which I had paid in. After a short time I felt that I was again able to pay into the Fund, and I therefore applied to the Secretary for a new policy; also for one for sick-pay. Both were granted, and although I have had to borrow a small sum from the Fund on two occasions, I have not had to take the whole of the money out again, and I have repaid the amount which I borrowed on my policy. I am quite sure that I should not have saved any money had I not been obliged to send it to the Pension Fund, for, like many other nurses, I think I often want things which I can do without. This year my health has not been good, and I have had to apply for sick-pay, and also to ask the advice of our kind medical referee, Dr. G. W. Potter, to whom my sincere thanks are tendered for the advice which he so courteously gave me, and the prescription which has done me so much good. The small sum from the sick-pay coming so regularly has helped me, with the amount which I had in hand, to tide over what would otherwise have been a very anxious time; and I am only too pleased to tell other nurses of the benefit the Fund has been to me. I can promise them the greatest courtesy from the Secretary and his staff, who are always ready to answer all questions, that those who join may know exactly what they have to pay and the benefit they will receive for their payments. It must be rather disappointing that so few nurses attend the annual meetings* of the Fund. The great and personal interest which Mr. Everard A. Hambro and all those who speak at that meeting take in the nurses and their welfare cannot be realised until one has been present at a meeting and has heard and seen for herself.

* NOTE.—The annual meeting will be held this year at River Plate House, Finsbury Circus, close to Moorgate Street Station, on Thursday, March 21 next.—ED. THE HOSPITAL.

### Appointments.

ADDENBROOKE'S HOSPITAL, CAMBRIDGE.—Miss Gregory has been appointed night sister. She was trained at Hertford Infirmary. She has done several years' private nursing, and has been staff nurse at Addenbrooke's Hospital since November 1905.

BELPER WORKHOUSE INFIRMARY.—Miss K. M. Underwood has been appointed superintendent nurse. She was trained at Birmingham Infirmary, and has since been theatre sister and maternity sister in the same institution.

BIRMINGHAM INFIRMARY.—Miss Shaw has been appointed sister. She was trained at Hertford Infirmary, and has since been staff nurse at Addenbrooke's Hospital, Cambridge.

CHICHESTER INFIRMARY.—Miss C. Bunting has been appointed night sister. She was trained at Addenbrooke's Hospital, Cambridge, and has since been theatre nurse in the same institution.

DARLINGTON HOTEL AND DISPENSARY.—Miss Miriam Ashley has been appointed lady superintendent. She was trained at the General Hospital, Birmingham, where she has since been Sister. She has also been night superintendent at the Royal Infirmary, Bradford, and assistant-matron at the Royal Halifax Infirmary.

LAMBETH POOR-LAW INFIRMARY.—Miss Band has been appointed sister. She was trained at Addenbrooke's Hospital, Cambridge.

LINCOLNSHIRE NURSING ASSOCIATION.—Miss Margaret M. White has been appointed superintendent of this Association, which is affiliated to Queen Victoria's Jubilee Institute. She received her district training under the Scottish branch in Edinburgh, being appointed Queen's nurse in July 1900; since that date she has held various posts as Queen's nurse in Scotland.

PRINCESS ALICE MEMORIAL HOSPITAL, EASTBOURNE.—Miss Bessie F. Potter has been appointed sister. She was trained at the Royal Surrey County Hospital, Guildford, and has since been staff nurse at the Hospital for Sick Children, Great Ormond Street, Bloomsbury.

READING INFIRMARY.—Miss Maud Emmeline Large has been appointed charge nurse. She was trained at Epsom Union Infirmary.

STAFFORDSHIRE COUNTY NURSING ASSOCIATION.—Miss Margarete Egestorff has been appointed superintendent of nurses to this Association, which is affiliated to Queen Victoria's Jubilee Institute. She was trained at Radcliffe Infirmary, Oxford, and for district nursing by the Metropolitan Nursing Association, Bloomsbury Square, London. She has since been sister at University College Hospital, London, and Queen's Nurse in Gower. She holds the C.M.B. certificate.

ST. MARY'S COTTAGE HOSPITAL, TENBURY.—Miss J. Hewett has been appointed matron. She was trained at the Metropolitan Hospital, Kingsland Road, N.E., where she was afterwards holiday sister. She has since been charge nurse at the Devon and Cornwall Homœopathic Hospital and Three Towns Dispensary, Plymouth; nurse and holiday matron at the West of England Nurses' Co-operation, Plymouth; and sister at the Royal Hospital for Incurables, Putney Heath. She has also done massage, electrical, and gynæcological work.

STROUD HOSPITAL.—Miss Edith Bruce has been appointed charge nurse. She was trained at the Royal Infirmary, Glasgow. She has since had charge of the women's ward of the Mildmay Memorial Cottage Hospital, and has been staff nurse at the Brighton Throat and Ear Hospital. She has also done district work.

### Midwives' Defence Union.

THERE was quite a crowded meeting at the Midwives' Institute on Friday last, in response to an announcement that a special discussion would be opened on the subject of a "Midwives' Defence Union." Dr. Atkinson took the chair, but the discussion was regarded as private until the aims and plans of the proposed Union can be more matured. Meanwhile a committee was appointed to inquire into the matter. At the close of the meeting the Chairman expressed a hope that when the Defence Union is fairly launched the Press will give all possible publicity to the matter, and that the nursing and midwifery papers will encourage correspondence on points of difficulty between town and country members.

### Death in our Ranks.

THE death of Miss B. C. Morris, a nurse at Wolverhampton Workhouse Infirmary, took place a few days ago, and at her funeral on Thursday last at Rugeley a number of the Wolverhampton Guardians, the medical officer, nurses, and other officials were present.

### The Nurses' Bookshelf.

THE NURSES' "ENQUIRE WITHIN." By C. O. M. (London: The Scientific Press, Limited. Pp. viii. and 166. Price 2s. net.)

THIS is a small pocket encyclopædia giving information about diseases, symptoms, and nursing treatment. It is arranged alphabetically so that readiness of reference is secured. There is no attempt to supplant the larger, systematic text-books on nursing; the proposal is to provide a reference book which can be kept always at hand and to which an appeal may be made in an emergency. The "Enquire Within," we think, fully justifies its own existence. It is well arranged and carefully and lucidly written. The least successful section is that dealing with "medicines." To speak about "drop doses" of strychnine, mercury, and sulphuric acid, is to run the risk of serious error. But in its special domain this book will, we are sure, have a large opportunity for usefulness.

### "The Hospital" Convalescent Fund.

THE Hon. Secretary begs to acknowledge with thanks the receipt of 2s. 6d. from L. B. Marsh and 2s. from Miss N. Garner.

### Wants and Workers.

WILL any kindly disposed person possessing a wheeling chair for which they have no further use be good enough to give it to a very poor girl, who has never walked from birth? At present her only means of locomotion consists in jerking herself about on an ordinary rocking-chair. Carriage would be gladly paid.—Address, Sister Thomas, Trevecca College, Talgarth, Breconshire.

### INFANTS' WEIGHT CHART.

THE price of the weight-chart brought out by W. H. Bailey and Son, Ltd., 58 Oxford Street, W., which we noticed last week, is 6d.

### Notes and Queries.

**REGULATIONS.**

**The Editor is always willing to answer in this column, without any fee, all reasonable questions, as soon as possible.**

**But the following rules must be carefully observed.**

**1. Every communication must be accompanied by the name and address of the writer.**

**2. The question must always bear upon nursing, directly or indirectly.**

**If an answer is required by letter a fee of half-a-crown must be enclosed with the note containing the inquiry.**

### *Massage.*

(210) Can you tell me of the nearest place to Doncaster where I can get massage training?—*Dodo.*

Write to the Incorporated Society of Trained Masseuses, 12 Buckingham Street, Strand, where you may obtain information.

### *Home for Aged Man.*

(211) Can you tell me of a home for a man of 69, who has a pension as workhouse master?—*Maidenhead.*

The Homes for the Aged Poor (apply to Misses Harrison, 86 Penge Road, Anerley, S.E.) might be suitable, or Nazareth Home, Hammersmith, might receive him when he is 70.

### *Nursing Administration.*

(212) Can you inform me if a Select Committee of the House of Commons or other authority has been recently convened to inquire into nursing administration? If so, where can I get the Blue-books?—*Oldham.*

A Committee of the House of Commons sat in 1891, and the results of this and the House of Lords Commission on Hospitals can be procured from Messrs. King and Co., Westminster. A good deal of evidence was elicited during the recent inquiry into the pros and cons of State Registration. You can probably also get this at King's.

### *Embrocation and Irritation.*

(213) (1) Would it be possible for Roche's embrocation to leave a rash and roughness on a child after using it only two or three times two years ago, when at the age of six months; or might the rash be from other natural causes? (2) If so, what can I do to get rid of the rash and roughness?—*M. H.*

(1) We think the irritation caused by some embrocations is likely to set up an eczema, which may become chronic. It is therefore dangerous to apply advertised embrocations to the delicate skins of young children. (2) Consult a medical man, or take the child to a hospital.

### *A Bed for a Case of Rheumatic Arthritis.*

(214) I venture to ask your advice upon a bed suitable for a severe case of "rheumatic arthritis." The patient has for some time been lying upon a water rubber bed, only filled with air, and last week was put upon the latest air-bed. But we find now that the pressure of the corrugation under the thighs causes very much pain and elevates the feet and legs to a higher level than that of the back.—*Pansy.*

Probably the bed has not been distended enough. It should be quite full and absolutely without leakage; otherwise wrinkles are sure to be made and the patient to sink down in the bed in a most uncomfortable condition. If the air-bed cannot be made comfortable better resort again to the water-bed.

### *Competition Prizes.*

(215) Can you tell me of the value of the prizes awarded to successful candidates answering the nursing questions in The Hospital. I have been competing with success."—*E. M. D.*

As you have been a successful competitor, you will before now have had a list of books with values, etc., sent to you.

### *Oxygen.*

(216) I should be glad to know how to administer oxygen.—*Inquirer.*

You must not do anything until you have been instructed by a medical man.

**Handbooks for Nurses.**

| | Post Free. |
|---|---|
| "A Handbook for Nurses." (Dr. J. K. Watson) ... | 5s. 4d. |
| "How to Become a Nurse." (Sir Henry Burdett) ... | 2s. 4d. |
| "Nurses' Pronouncing Dictionary of Medical Terms" | 2s. 0d. |
| "Art of Massage." (Creighton Hale) ... ... ... | 6s. 0d. |
| "Surgical Bandaging and Dressings." (Johnson Smith.) ... ... ... ... ... | 2s. 0d. |
| "Surgical Instruments and Appliances Used in Operations," illustrated. (Harold Burrows, F.R.C.S.) | 1s. 8d. |
| "Hints on Tropical Fevers." (Sister Pollard.) ... | 1s. 8d. |

Of all booksellers or of The Scientific Press, Limited, 28 & 29 Southampton Street, Strand, London, W.C.

### For Reading to the Sick.

"BE THOU NEAR US."

From darkness here, and dreariness,
We ask not full repose :
Only, be Thou at hand, to bless
Our trial hour of woes :
Is not the pilgrim's toil o'er-paid
By the clear rill and palmy shade?
And see we not, up Earth's dark glade,
The gate of Heaven unclose?

*Keble.*

The Holy Ghost is given; we may be partakers of His Presence; our life may be a training, a renewing, a carrying out of His work; every temptation may be to us as the graving of the Hand of God. In prayers and watchings, in self-denials and aspirations, in blessings received meekly and used thankfully, in pains and griefs borne without repining and made by patience as steps upwards; in all of these a life of God may in very truth be ours. God may be, will be, unless we will it not, working upon us, re-making us, fitting us for the open vision and the full enjoyment of Himself.—*Bishop S. Wilberforce.*

Sharp suffering is so separating a thing, it does so truly set the sufferer alone, that it is oftentimes too strong for merely human sympathy. The stricken will be the lonely hearted; but from His sympathy, from the unseen but most real visitations of His blessed Spirit, it cannot part us. Nay, it is to the faithful a living discipline, which wounds indeed for the present their aching senses, but which more than anything besides brings them near to Him. They taste the fellowship of the Man of Sorrows. Their anguish is as a wall built up between them and others, but within that wall, as into a shrine, He will come Whose Presence is all joy. On Him, too, are the abiding marks of a like affliction. Pierced Hands and a wounded Side; these are the tokens of His fellowship with sufferers; and still, as of old, to those whom He has withdrawn into that same Sanctuary, He showeth His Hands and His Feet, until they are ready "to believe not for joy, and to wonder" at the grace vouchsafed unto them.—*Ibid.—Bishop S. Wilberforce.*

Surely this should be the law of your life, the fixed habit of your soul. If union with Him be true, in trust, in love,—the thought that He knows all, that He is present, that He comes with special grace to heal, to soothe, to support, apprehended by a vivid act of faith, would have power to sustain the soul, would grow to be a settled support, a stay in the midst of every trouble, a sufficiency of strength, a power against inward disquiet and what remains of self to drag down the spirit.—*T. T. C.*

Thrice bless'd are they, who feel their Loneliness;
To whom nor voice of friends nor pleasant scene
Brings aught on which the sadden'd heart can lean.
Yea, the rich earth, garb'd in her daintiest dress
Of light and joy, doth but the more oppress,
Claiming responsive smiles and rapture high,—
Till, sick at heart, beyond the veil they fly,
Seeking His Presence Who alone can bless.

*Newman.*

